# The Story of the Insane from Year to Year

**Published:** 1902-07-26

**Authors:** 


### THE STORY OF THE INSANE FROM YEAR TO YEAR.

FOURTEENTH ANNUAL SERIES.

### HEREFORD COUNTY ASYLUM.

ON January 1st this asylum contained 367 patients, and on December 31st the number had risen to 378. There were 64 first admissions, 23 readmissions, 31 discharged recovered, 5 discharged relieved, and 32 died. The average number resident was 377, and the total number under treatment was 448. The recoveries for the year stand at 37·9 per cent. of the admissions, and the deaths at 8·4 per cent. of the average number resident. The former is about the average of kindred institutions, and the latter is below the average. Of the deaths 10 were caused by cerebral and spinal diseases, and there was no death from consumption. Turning to Table X., we find that moral causes account for the insanity in 7 of the year's admissions, and among the physical causes, intemperance in drink was the cause in 13 instances and hereditary influence in 7. Dr. Morrison deplores the influx of incurable senile cases, and says it is a prominent and depressing feature of this asylum. There is no doubt a tendency in the Poor Law Unions to transfer cases from the workhouses to the asylum—cases which could quite well be kept in these workhouses—and the cause has been pointed out over and over again, but nothing has been done to stop it. To us the evil seems to lie in two directions: first there is the unnecessary expense of asylum treatment; and second, there is the ever-recurring enlargements of the asylum to accommodate these chronic incurables. But we join issue with Dr. Morrison when he says that the presence of "such a mass of chronic, incurable, senile cases imperils the keen individual attention needful for the recovery of the acute recoverable cases." And he adds "that every failure at recovery adds to the permanent charge the ratepayer has to meet." Here, again, we think he is wrong. Had he said that a medical man was bound to use every lawful means to ensure the recovery of his patient he would have been right; but nothing can be more certain than that every cure made in the Hertford Asylum (unless it be in women over the age of 40) is very likely to increase the permanent charge on the rates, and not to diminish it. Dr. Morrison's report is, however, an unusually interesting one, and his remarks on alcoholic insanity are extremely good. "The natural enemy of all excess," he says, "should be the schoolmaster. The increase of alcoholic insanity with a higher wage is not incidental to this country alone, but observable generally." Too true is this, we fear. So with the artificial warming of the air which patients are compelled to breathe; and we believe that if Dr. Morrison will further study and experiment on this question he will be confirmed in his ideas, and we hope his next report will inform us that he has entirely cut off that poisoned stuff from his dormitories at any rate. The weekly rate of maintenance is 10s. 2½d. per head.

SURREY COUNTY ASYLUM AT BROOKWOOD.

HERE the numbers were 999 on January 1st and 1,050 on December 31st. There were 239 first admissions, 46 readmissions, 89 recoveries, 23 discharged relieved, and 101 deaths. The average number resident was 1,033, and the total number under treatment was 1,284. The recoveries were 32·85 per cent. of the admissions, and the deaths 9·77 per cent. on the average number resident. Of the deaths 46 were caused by cerebral and spinal diseases, 14 by consumption, and 12 by other lung diseases. Of the year's admissions the insanity was in 77 cases accounted for by such moral causes as domestic troubles, adverse circumstances, mental anxiety, religion and love, while among the physical causes hereditary influence heads the list with 57, previous attacks come next with 51, and intemperance in drink follows with 35. After 26 years' service Nurse Marshall received a pension of two-thirds of her salary and allowances. A third assistant medical officer has been appointed at a salary of £120 a year, and the Commissioners "hope that it will now be found possible to undertake some of the finer pathological work now so usual in asylums." The committee express their "approval of the kind and careful way in which the asylum has been carried on by the able superintendent, Dr. Barton, and his assistants." The weekly charge to the Unions was 11s. per head.

STAFFORDSHIRE ASYLUM AT CHEDDLETON, NEAR LEEK.

THIS asylum issues its second annual report, and from it we learn that it contained 296 patients on January 1st, and on the last day of December the numbers had risen to 611. There were 413 first admissions, 3 readmissions, 21 recoveries, 10 discharged relieved, and 60 deaths. The average number resident was 448, and the total number under treatment was 709. As something more than 200 of the admissions were transfers from other counties it would be useless to give percentages of recoveries and deaths, as such figures would be valueless for purposes of comparison with other asylums. We notice, however, that colitis was the cause of death in four cases, and that there were 29 cases of this disease. Dr. Menzies says that the "high percentage need never occur again, for we can remove the first cases to the infectious hospital and prevent the bed-to-bed spreading which here occurred." This new asylum has followed some of the older ones in employing nurses of a better social position than is usual in the majority of asylums, and Dr. Menzies tells us that his highest hopes have been realised, and he adds that "the only difficulty is a lack of candidates which he never anticipated." We have more than once in these notices expressed our surprise that so many candidates are ever knocking at the doors of the hospitals when many medical superintendents find a difficulty in keeping up the numbers of their nursing staffs, and it is a fact that in many ways the asylum nurse is better off than the hospital nurse. The asylum has 110 patients from other counties, and these are charged 15s. a week each. The charge for home patients is 9s. 6d.

WORCESTER COUNTY ASYLUM.

ON January 1st this asylum contained 1,146 patients, and on the last day of the year the number had increased to 1,169. There were 216 first admissions, 31 readmissions, 79 recoveries, 8 discharged relieved, and 128 deaths. The total number of patients under care during the year was 1,387, and the average number resident was 1,144. The recoveries stand at 32·7 per cent. of the admissions, and the deaths at 11·1 per cent. of the average number resident. The former percentage is lower and the latter higher than the County Asylum averages; and they are respectively lower and higher than the Worcester average. Of the year's deaths 35 were caused by cerebral and spinal diseases, 26 by consumption, 26 by pneumonia, 3 by pleurisy, and 1 by colitis. Of the year's admissions 12 were supposed to owe their insanity to various moral causes, and of the physical causes drink was noted in 24 cases, hereditary influence in 27, and some family predisposition in 37. Several minor accidents happened during the year, including 4 cases of fractured ribs, 1 of the femur, and 1 of the lower jaw. There was 1 fatal case of enteric fever, and the source of the infection could not be traced. Such isolated cases are by no means rare in our county asylums, and they will occur in patients who had not been beyond the grounds for years and have had no communication with the outside world; hence the most careful investigation fails to account for the outbreak of the disease. Colitis was also present during the year. The Visiting Committee state that the medical superintendent and the assistant medical officers continue to discharge their several duties to their satisfaction. The weekly charge to the Unions is 7s. 7d. per head.

